# Crosstalk between stem cell and spinal cord injury: pathophysiology and treatment strategies

**DOI:** 10.1186/s13287-019-1357-z

**Published:** 2019-08-06

**Authors:** Anwen Shao, Sheng Tu, Jianan Lu, Jianmin Zhang

**Affiliations:** 1 Department of Neurosurgery, Second Affiliated Hospital, School of Medicine, Zhejiang University, Hangzhou, 310003 China; 2 Department of Infectious Diseases, Collaborative Innovation Center for Diagnosis and Treatment of Infectious Diseases, The First Affiliated Hospital, School of Medicine, Zhejiang University, Hangzhou, 310003 China; 3 Brain Research Institute, Zhejiang University, Hangzhou, 310003 China; 4 Collaborative Innovation Center for Brain Science, Zhejiang University, Hangzhou, 310003 China

**Keywords:** Spinal cord injury, Stem cell, Mechanism, Therapy, Review

## Abstract

The injured spinal cord is difficult to repair and regenerate. Traditional treatments are not effective. Stem cells are a type of cells that have the potential to differentiate into various cells, including neurons. They exert a therapeutic effect by safely and effectively differentiating into neurons or replacing damaged cells, secreting neurotrophic factors, and inhibiting the inflammatory response. Many types of stem cells have been used for transplantation, and each has its own advantages and disadvantages. This review discusses the possible mechanisms of stem cell therapy for spinal cord injury, and the types of stem cells commonly used in experiments, to provide a reference for basic and clinical research on stem cell therapy for spinal cord injury.

## Introduction

Spinal cord injury (SCI) is currently the most difficult traumatic neurological condition to treat in the clinic. Following the primary injury, which causes immediate structural damage, a series of secondary injuries, including hemorrhage, edema, demyelination, and axonal and neuronal necrosis, are involved in the pathological process after SCI [1, 2]. Afterwards, a fibrous glial scar formed by infiltrated inflammatory cells, including microglia, fibroblasts, and reactive astrocytes, limits axon regeneration across the lesion [3, 4]. Strategies targeting these unique mechanisms, as well as neuroprotective and regenerative therapies, are expected to be used as treatments for SCI. Neuroprotective therapy works by limiting secondary damage, while neuroregenerative strategies aim to replace the damaged cells, axons, and circuits in the spinal cord [5]. Although few neuroprotective and regenerative therapies that directly exert beneficial effects are currently available [6], cell therapies with neuroprotective effects and neuroregeneration potential may represent a new horizon in the treatment of SCI. Since Orlic et al. [7] first performed stem cell transplantation for coronary heart disease in 2001, stem cell transplantation has been widely employed for the treatment of different diseased tissues and organs. Although the biological characteristics of various types of stem cells differ, the therapeutic effects of stem cells that are recognized by the current research are mainly manifested in three aspects. First, stem cells have their own multidifferentiation potential and play a role in replacing degenerative necrotic cells. In addition, stem cells secrete anti-inflammatory factors that inhibit the inflammatory reaction in the damaged microenvironment. Finally, stem cells produce many cytokines, growth factors, and cell adhesion factors that play important roles in improving the microenvironment and promoting tissue regeneration [8–10]. Based on these characteristics, stem cell therapy is considered the most promising treatment in regenerative medicine. In recent years, with the advent of in-depth research of stem cell biology and translational medicine, the use of stem cell transplantation and stimulation of potential stem cell differentiation in vivo to treat irreversible dysfunction caused by SCI has achieved remarkable results [11, 12]. Although stem cell transplantation for SCI is currently the most promising treatment used in neuroregenerative medicine, the biological characteristics and physiological functions of different types of stem cells vary (Table 1). We reviewed the research progress that has recently been achieved in applying these stem cells to treat SCI.

**Table 1:** Source, definition, mechanism, advantage, and current limitation of stem cells in SCI

	Description	Possible therapeutic effects in SCI	Advantages	Current limitation
Mesenchymal stem cell	Mesodermal lineage multipotent progenitors can be obtained from bone marrow, umbilical cord, amnion, placenta, and fat tissue [13].	Secreting anti-inflammatory factors, cytokines, growth factors, and cell adhesion factors to improve the microenvironment of the lesion and further promotes self-repair after SCI; immunomodulatory, neurotrophic and anti-apoptotic effects [13, 14].	High multilineage differentiation, easily isolated and grafted, suitable for different stages of SCI, raising no ethical concern, limited risk of developing tumors, minimal immunoreactivity [15, 16].	Mechanism requires further research which limits the efficiency of treatment; results of clinical trials are still far from obtaining functional recovery and restoring neural circuits; effective way to deliver cells still needs further research [16].
Embryonic stem cells	Highly undifferentiated cells that are pluripotent and can differentiate into different tissue cells [17].	Differentiated neurons and glial cells are used to supplement cell defects caused by SCI; secrete active factors to inhibit further damage, support nerve tissue regeneration [18–23].	Long history of research, proven to have a certain effect in a variety of diseases; pluripotent cells that can differentiate into all tissue cells [17, 18].	Immune rejection and the risk of tumor formation; ethical issues needed to be solved [24–26].
Neural stem cells	Stem cells located in the lateral ventricle of the brain, the dentate gyrus of the hippocampus, and the central canal of the spinal cord [27].	Modulation of the formation of glial scar, enhancing oligodendrocyte differentiation and neuronal differentiation; replace necrotic damaged cells and reconstruct local loops which in turn promotes the recovery of body function; secrete growth-promoting factors to promote the survival and growth of damaged neurons; immunomodulatory effects [28–32].	A reducing tumorigenicity due to the maturation is restricted to glial and neuronal subtypes; can be harvested from either adult or fetal spinal cord tissue [33, 34].	Further studies are necessary to confirm neurological and functional benefits, safety, adjusting doses and administrations periods as well as the most promising cellular sources to obtain NSCs [34].
Induced pluripotent stem cells	Considered to be effective alternative cell sources for ESCs.	Induced to be neural progenitor cells, neurons, oligodendrocytes, and astrocytes; promote remyelination, axonal regeneration and the secretion of neurotrophic factors; reducing inflammation [35].	Self-renew and differentiate into various types of neural cells; Free of ethical issues associated with some transplant sources and importantly can be performed in an autologous manner removing the need for immune suppression [36].	High risk of immune rejection and tumorigenesis: teratoma and true tumors [13, 36].
Spermatogonial stem cells	A subtype of spermatogonia [37].	Have the potential to differentiate into various cells nervous system including functional GABAergic neurons, glutamatergic neurons, serotonergic neurons, and glial cells [38, 39].	Multidifferentiation potential; self-replication and self-renewal abilities; able to differentiate into functional dopaminergic neurons directly without an intermediate transition process of ESCs or NSCs; pass the genetic material to the offspring; can be produced throughout the lifetime and lacking of ethical problems, tumorigenicity and immune rejection [37, 40–43].	Multi-differentiation potential is susceptible to environmental influences; SSC treatment of nervous system diseases is exclusively designed to substitute for differentiation at present, and no reports of secretion of various factors by these cells have been found [42].
Adult endogenous stem cells	Stem cells located in the adult nervous system [27].	Activate and proliferate to produce glial cells when spinal cord is injured; differentiate into astrocytes and oligodendrocytes [27, 44, 45].	Noninvasive cell therapy that directly activated to function without the need for traumatic cell transplantation.	Ability to differentiate into neurons is limited [46].

### Pathological period of SCI and stem cell therapy strategy

The pathological period of SCI is divided into three phases: acute phase (< 48 h), sub-acute phase (48 h to 14 days), and chronic phase (> 6 months) [47]. Since each characteristic pathophysiological period is different, the appropriate cell therapy must be selected according to these unique conditions. After the primary injury, the first period is the acute phase. During this period, due to hemorrhaging, the infiltration of inflammatory cells, cell necrosis and the release of cytotoxic products, a strong inflammatory reaction will occur in the injured area [48]. Therefore, an anti-inflammatory treatment is particularly important in the acute phase [49]. In addition to inflammatory, oxidative responses also play a contributory role in the pathogenesis of the secondary damage after SCI [11, 50]. During oxidative stress, molecular oxygen is inadequately reduced in the mitochondria, resulting in excessive levels of ROS (reactive oxygen species) [51]. ROS can then lead to various types of destructive effects such as lipid peroxidation, DNA damage as well as cell death [52]. Antioxidant therapy attenuates oxidative stress injury in the acute phase, which in turn contributes to the recovery of neurological function after SCI [53–55]. The sub-acute phase follows the acute phase, during which the cystic cavity begins to coalesce, and damaged and denuded axons degenerate or is retracted. During this period, treatments regulating axon growth will promote neuritis elongation [6, 56]. In the chronic phase, axonal regeneration is limited. Therefore, remyelination will play a protective role in this period [47]. In addition, strategies designed to strengthen the correct neural circuits and synapse formation also help to promote recovery in the later stages of SCI [57]. Embryonic stem cells (ESCs), neural stem cells (NSCs), mesenchymal stem cells (MSCs), and oligodendrocyte precursor cells (OPCs) promote neuroprotection in a manner specific to each treatment mechanism. MSCs, NSPCs, and Schwann cells are frequently reported to function by secreting bioactive molecules such as trophic factors and cytokines. ESCs, NSCs and MSCs are usually used to accelerate axonal regeneration and growth; myelin regeneration is closely related to oligodendrocytes derived from NSCs or OPCs [58]. In addition, many other stem cells with different functions have been used for therapeutic purposes.

### Neural stem cells

Neural stem cells (NSCs) are stem cells located in the lateral ventricle of the brain, the dentate gyrus of the hippocampus, and the central canal of the spinal cord. As a barrier to the brain and spinal cord parenchyma, they are closely related to cerebrospinal fluid circulation [27]. The main mechanism of the therapeutic effects of NSCs on neurological diseases includes the modulation of the astrocytes contribution to the glial scar, enhancing oligodendrocyte differentiation and neuronal differentiation [28], replacing the missing nerve cells in SCI and secreting pro-regenerative factors to protect damaged tissue cells and neuritis [59, 60]. Currently, the application of NSCs in the treatment of SCI animal models is mainly focused on the spinal crush injury model [61]. The transplantation of NSCs into the damaged spinal cord tissue effectively promotes the recovery of body function. On the one hand, NSCs entering the injured area differentiate into neurons, which directly replace the lost neurons or provide a neuronal substrate for electrical signals to bridge or circumvent the lesion area [28, 62]. Injured axons after SCI can form connections with the transplanted NSCs, creating a relay circuit that may potentially bridge the disrupted tracts [1, 63]. Axonal growth at the injury site is significantly accelerated, and axonal conduction is modestly improved after transplantation of NSCs [29]. On the other hand, NSCs secrete a large number of growth-promoting factors [BDNF, CNTF, GDNF, NGF, insulin growth factor-1 (IGF-1), etc.] to promote the survival and growth of damaged neurons [30]. An important feature observed following SCI is demyelination; thus, by promoting oligodendrocyte differentiation, NSCs enhance myelination and improve motor and sensory function when transplanted into the injured spinal cord [31]. The formation of a glial scar is closely related to the differentiation of NSCs. Without the scar component from NSCs, the depth of the SCI progressively increases over time, implying that NSCs function as a scaffold within the scar to restrict the secondary enlargement of the lesion and prevent the lesion from expanding after the initial insult [64]. According to a previous study [65], the therapeutic effects of NSC transplantation include immunomodulatory effects, such as the regulation of T cells and macrophages to inhibit inflammatory demyelination. Under these conditions, the main role of NSCs is to reduce the number of CD4+ T cells and shift microglia cells change from a harmful to beneficial phenotype, and thus the treatment of SCI by NSCs may be mediated by a combination of multiple factors. Several clinical trials of NSCs are ongoing, with early results showing segmental and several sensory improvements below the injury level. Studies published to date have shown that NSC transplantation for SCI is relatively safe, but whether it effectively improves the patient’s function after transplantation remains controversial [32]. The phase II trials by Stem Cells Inc. (Newark, California) of human CNS stem cell transplants for cervical (*n* = 31; NCT02163876) and thoracic (*n* = 12; NCT01321333) injury showed that the sensory function of some patients with a mild injury was restored [5], and no increase in treatment-related complications was observed [33]. Although this study was terminated in 2016 due to an unknown reason, it certainly provided a good foundation for the future application of NSCs. Although the research on NSCs has achieved substantial progress, the combination of NSCs and other stem cell therapies might produce better therapeutic effects.

### Mesenchymal stem cells

Mesenchymal stem cells (MSCs) have become the favorite seed cells in the preclinical and clinical practice of regenerative medicine due to their readily obtainable source, wide biological effects, lack of ethical problems, and low immunogenicity [13]. A large number of MSCs have been obtained from tissues such as bone marrow, umbilical cord, amnion, placenta, and adipose tissue [12]. Although the characteristics of MSCs from different tissue sources differ, MSCs have shown good therapeutic efficacy in the treatment of various central nervous system (CNS) diseases [66–68]. According to previous studies [14, 69, 70], the transplantation of MSCs into animal models of Alzheimer’s disease, stroke, Parkinson’s disease, multiple sclerosis, and lateral sclerosis effectively ameliorated the symptoms of the animals. Furthermore, this effective therapeutic effect is related to the anti-inflammatory, immunomodulatory, neurotrophic, and anti-apoptotic effects of MSCs [13, 14]. The mechanism underlying the biotherapeutic action of MSCs is mainly that after MSCs enter the lesion, a large number of anti-inflammatory factors, cytokines, growth factors, and cell adhesion factors are released by paracrine signaling, which improves the microenvironment of the lesion and further promotes self-repair by these cells, whereas its differentiation and substitution effects are not obvious [14]. Although the mechanism of this treatment requires further research, MSCs have shown good application prospects in the treatment of various diseases.

#### Bone marrow mesenchymal stem cells

Bone marrow mesenchymal stem cells (BM-MSCs) are derived from bone marrow but are different from bone marrow hematopoietic cells, which grow in an adherent manner and differentiate into mesoderm cells. Currently recognized markers that are specific for BM-MSCs include CD29, CD90, and CD44 [69]. Initial studies have revealed the strong differentiation potential of BM-MSCs into osteoblasts, chondrocytes, chondroblasts, adipocytes, fibroblasts, and different subtypes of neurons and glial cells under different induction conditions [70, 71]. Therefore, BM-MSCs are expected to be an ideal candidate for the treatment of SCI. In addition to the use of rodent and human BM-MSCs, current preclinical studies have examined the therapeutic effects of primate and porcine BM-MSCs on SCI. The motor function of the injured animals was significantly improved when BM-MSCs were transplanted into the contused, crushed, or transverse wounded spinal cords [24]. The main BM-MSC transplantation methods that are currently used include in situ injection, intravenous injection, intrathecal injection, or intraventricular injection [24, 72, 73], but the preclinical experimental methods are mainly orthotropic transplantation into the injured spinal cord [49]. To date, BM-MSCs have been shown to promote spinal cord regeneration through three main mechanisms. First, BM-MSCs exert an immunosuppressive effect, protect against inflammatory reactions, and inhibit lymphocyte proliferation and differentiation in the SCI region. Second, BM-MSCs in the injured area promote the transition of M1 macrophages to the M2 type. In addition, BM-MSCs release a large number of growth factors that protect the damaged spinal cord tissue from further damage [74, 75]. Among the trophic factors secreted by BM-MSCs, vascular endothelial growth factor (VEGF), nerve growth factor (NGF) and glial-derived neurotrophic factor (GDNF), and brain-derived neurotrophic factor (BDNF) have been identified. In addition, some growth factors, such as neurotrophin-3 (NT-3), fibroblast growth factor (FGF), and epidermal growth factor (EGF), have also been identified [52]. These factors support growth and promote neuritis regeneration [53, 54]. Based on these characteristics, BM-MSCs promote the regeneration of damaged spinal cord and improve the motor function of the animal to some extent [53]. Although these factors are important, the amount secreted by BM-MSCs is usually limited. The expression of these factors must be increased to significantly increase the regeneration of the damaged spinal cord. Currently, researchers generally use BM-MSCs as a release vector to introduce neurotrophic factor genes such as NT-3, GDNF, and BDNF, and then transplant them into the injured area of the spinal cord, further enhancing neuritis regeneration and the recovery of neurological function [55]. A large number of preclinical studies have confirmed that this treatment strategy is safe and effective [56]. Nevertheless, some clinical e studies have not observed a significant therapeutic effect after BM-MSC transplantation, mainly due to the lack of optimal cell transplantation conditions and transplantation times [57]. In situ transplantation and intravenous injections may cause different numbers of cells to reach the injured area. In clinical practice, in situ transplantation is usually not used to avoid secondary injury, resulting in a difference in the therapeutic effect. For these reasons, the clinical practice of cell transplantation using intravenous injection requires further exploration.

#### Adipose-derived mesenchymal stem cells

Adipose-derived mesenchymal stem cells (A-MSCs) are derived from adipose tissue and can be obtained from liposuction and liposuction. The advantages of A-MSCs are that large numbers of cells are easy to obtain, result in fewer traumatic injuries, are not associated with ethical problems, and can be autologously transplanted. The mechanism by which A-MSCs treat SCI and promote regeneration is by secreting a large number of neurotrophic factors, such as BDNF and GDNF, regulating activated immune cells and promoting nerve regeneration and anti-apoptotic effects [76–78]. In addition to these biological effects, A-MSCs also possess a multilineage cell differentiation potential and differentiate into adipose-derived, osteogenic, cartilage-derived, myogenic, smooth muscle-derived, neurogenic, and endothelial-derived cells, as well as Schwann cells [79]. This multidifferentiation potential also confers A-MSCs with the potential to exert regenerative effects on SCI by replacing/supplementing nerve cells that are degenerated or undergoing necrosis in SCI through directed differentiation. Molecules secreted by A-MSCs also include growth factors, extracellular matrix molecules, proteases, cytokines, and immunomodulatory molecules [79]. These molecules play a key role in promoting angiogenesis and wound healing, assisting in the growth of new tissues, reducing inflammatory responses, and activating lymphocyte proliferation [79]. Based on these findings, a growing number of clinical studies and practices favor the treatment of SCI through A-MSC transplantation. Kolar et al. [78] transplanted A-MSCs into rats with a cervical spinal cord injury and observed the inhibition of glial scar formation and axonal regrowth, but unfortunately, rat forelimb function was not restored. In another study [79], A-MSCs were directly transplanted into the parenchyma of the spinal cord, and after 7 weeks, the animal’s body function was restored. A morphological examination showed the accumulation of a large amount of laminin and protrusions at regenerating axons located at the transplant site, and the tissue damage observed after SCI did not expand further. Similar results were reported in the study by Kim [80], who injected A-MSCs into the fourth ventricle of dogs with acute SCI and observed a significant improvement in hind limb movement with no side effects. Many scholars have begun to adopt a comprehensive treatment plan to further enhance the survival and treatment effect of A-MSCs at the transplant site that combines some molecules with A-MSCs to treat chronic SCI, such as adding 17β-estradiol to improve A-MSC-mediated secretion of expressed growth factors, overexpression of the BCL-2 gene to inhibit apoptosis, and simultaneously administer chondroitin sulfate proteoglycan-degrading enzymes to destroy keratinous scars [81–83]. The efficacy of these comprehensive treatment regimens in promoting functional recovery in animal models is significantly better than the A-MSC treatment alone. According to current preclinical studies and some clinical practices, A-MSC transplantation is safe and has no side effects [84].

#### Amniotic epithelial mesenchymal stem cells

Amniotic epithelial mesenchymal stem cells (AE-MSCs) are obtained from the amniotic membranes and amniotic fluid derived from embryos. The traditional hypothesis is that the amniotic membrane protects embryos and maintains the normal development of organs. It is often discarded as biological waste after maternal delivery. A large number of studies conducted in recent years have shown that AE-MSCs are also very effective MSCs for the treatment of SCI in regenerative medicine. They have many characteristics of MSCs and have therefore been used as seed cells for SCI regeneration [85]. The use of AE-MSCs for spinal cord regeneration is based primarily on their cytological properties, namely, multi-differentiation potential, strong proliferative capacity, growth factor production, lack of tumorigenicity and low immunogenicity. In some preclinical studies, the transplantation of parental AE-MSCs to treat offspring with SCI exerted almost no side effects and improved the symptoms of animal models of SCI [77, 86]. Bottai et al. [87] transplanted AE-MSCs into contused SCI model rats and found that hind limb motor function defects were significantly improved in animals. Results of the morphological analysis also showed a significant inhibition of myelin loss in the spinal cord of transplanted animals, the presence of a large number of new blood vessels in the injured area, and a significant decrease in the number of inflammatory cells moving into the injured area. The formation of blood vessels in the injured area is closely related to the cytokines and hepatocyte growth factor produced by AE-MSCs, revealing the unique character of AE-MSCs. The anti-inflammatory and anti-apoptotic effects of AE-MSCs are more effective in the treatment of SCI to promote motor function recovery, but AE-MSC transplantation combined with methylprednisolone is more effective than AE-MSCs or methylprednisolone alone [88], suggesting that methylprednisolone may exert a synergistic effect with the biological function of AE-MSCs. In addition, AE-MSCs have been transplanted into the cavity created by the spinal cord transection of monkeys, and AE-MSCs supported the entry of the regenerating processes into the cavity [89]. In summary, AE-MSCs effectively promote SCI repair mainly through trophic factor secretion to support growth and reverse the damage in the microenvironment and subsequently achieve nerve regeneration and the recovery of body function.

#### Umbilical cord mesenchymal stem cells

Umbilical cord mesenchymal stem cells (UC-MSCs) are MSCs derived from the umbilical cord or cord blood, which are easy to obtain and expand in vitro. The absolute advantage compared with other types of stem cells is their low immunogenicity. Many preclinical studies have found that transplantation of UC-MSCs into rodents with SCI significantly improve functional defects [58]. The therapeutic mechanism is mainly attributed to a combination of multiple factors, namely, the neurotrophic, anti-inflammatory, anti-apoptotic and pro-angiogenic effects of UC-MSCs [7, 27, 28]. Cytokines and trophic factors produced by UC-MSCs include interleukin-1 (IL-1), IL-10, neutrophil activator, NT-3, BDNF, VEGF, basic fibroblast growth factor (bFGF), and neural cell adhesion molecule (NCAM) [13, 86, 90]. Whether in animal experiments or clinical practice, orthotopic cell transplantation is more effective than an intravenous injection [13, 91], for similar reasons as described for the BM-MSC treatment mentioned above [13]. To date, few reports on the safety and efficacy of UC-MSC transplantation as a treatment for SCI have been published. In 2005, Kang et al. [92] transplanted human UC-MSCs into the lesion area of the injured spinal cord of a 37-year-old patient with SCI, and cell transplantation treatment continued for 41 days. Afterwards, the patient’s motor and sensory function improved significantly. After 1 year of follow-up, UC-MSC transplantation was safe for SCI. In 2013, Yao et al. [93] transplanted UC-MSCs into 25 patients with SCI using an intrathecal or intraventricular injection. Patients’ autonomic and somatic sensations recovered to varying degrees at 12 months after surgery. Different hypotheses for the therapeutic effect of UC-MSCs on SCI have been proposed. Zhu et al. [94] directly injected UC-MSCs into 28 lesions of the injured spinal cord and, combined with motor function training, found that the UC-MSC treatment did not cause serious adverse reactions. Fifteen patients achieved an effective motor function improvement, 12 patients displayed a partial improvement, and 1 patient did not display an effect of the treatment. Based on the results from these clinical studies, the efficacy of UC-MSCs as a treatment for SCI may be related to individual factors, such as the patients with SCI, transplantation methods and viability of UC-MSCs.

### Embryonic stem cells

Embryonic stem cells (ESCs) are highly undifferentiated, pluripotent cells that are able to differentiate into the cells of various tissues in adult animals. Because of their high degree of undifferentiation, these cells have been used as an in vitro cell differentiation and regulation model. Since Evans and Kaufman [17] first isolated and cultured mouse ESCs in 1981, research in this area has achieved rapid development in the past 20 years, particularly in the area of which ESCs are a good substitute for cell and gene therapy vectors. Currently, ESCs have been used to treat many diseases, including the repair of nerve damage and neurodegenerative diseases. Since ESCs are pluripotent cells that differentiate into all tissue cells, including neurons, they are also considered a highly potent neuronal cell replacement in the treatment of neuronal diseases [18]. The application of ESCs to treat SCI is mainly to use these differentiated neurons and glial cells to reverse the cell defects caused by SCI, and combined with the secretion of active factors to inhibit further damage, support nerve tissue regeneration, and ultimately achieve therapeutic and repair purposes [18]. The transplantation of predifferentiated ESCs into rat and mouse SCI models significantly improves motor dysfunction in the animals. Notably, Harper et al. [19] applied retinoic acid and sonic hedgehog (SHH) to induce ESCs to differentiate into motor neurons, and then transplanted the cells into the spinal cord of hemiplegic rats. The authors observed a significant improvement in the symptoms of hemiplegia in animals. Iwai et al. [20] also induced ESCs to differentiate into neural stem cells and then transplanted them into the injured spinal cord of monkeys. As a result, monkey motor function was significantly improved, and morphological evidence showed that myelin and axons in the spinal cord of animals transplanted with ESCs remained relatively intact. Yang et al. [21] also transplanted pig-derived ESCs into rats with spinal cord contusions and found that transplanted cells differentiated into neurons; behavioral tests showed significant improvements in motor function. In addition, ESCs expressing NT-3 and PDGF gene therapy vectors were transplanted into damaged spinal cord tissue. The ability of ESCs to differentiate into neurons and the numbers of surviving neurons were significantly increased [22]. ESCs have also been differentiated into oligodendrocytes to reconstitute the white matter as a treatment for SCI in another study [30]. By differentiating ESCs into functional OPCs, the cells were used to enhance remyelination after SCI [23]. Currently, among the many SCI cases in the world, many patients present with white matter destruction due to contusion. Therefore, the application of ESCs to differentiate into oligodendrocytes that reconstitute the white matter to achieve spinal cord repair and restore function is also the focus of current research. In addition to differentiating into corresponding cells, ESCs also secrete factors that enhance the phagocytosis of myelin debris and reduce lipid accumulation, as well as promote the conversion of microglia into an M2-like state to regulate the inflammatory response in the injured spinal cord, thus ultimately improving locomotor recovery [95]. In the study by Salewski RP et al., in addition to the conventional function induced by the differentiation of ESCs into the cells that maintain tissue integrity, the NSCs induced by ESCs maintain the regular shape of the gray matter and contour of the cord, indicating that a more complex mechanism may underlie the protective effect [96]. Although the clinical application of ESCs is promising, the future clinical application still faces enormous challenges due to ethical issues. The first problem that must be solved before ESCs are applied in the clinic is ethical issues. Additionally, because ESCs have the risk of tumorigenicity, rather than being transplanted in an undifferentiated form, they are usually initially induced to differentiate into neural precursor cells (NPCs) or OPCs, which are then transplanted into the body [5, 97, 98]. Currently, some clinical trials on NSCs have been conducted. Geron Corporation has conducted the first clinical phase I trial in 2009, which was terminated due to funding problems. Asterias Biotherapeutics has also recently initiated a phase I/IIa clinical trial in which OPCs derived from human ESCs are being used. These trials will make a significant contribution to the future use of ESCs in clinical practice.

### Adult endogenous stem cells

Adult endogenous stem cells (AESCs) are stem cells located in the adult nervous system. In the spinal cord tissue, ependymal cells, radial glial cells, and strip cells near the central canal of the spinal cord have stem cell characteristics [27]. Normally, these cells are dormant. Once the spinal cord is damaged, these cells are rapidly activated and proliferate to produce a large number of glial cells [27, 44, 45]. Sabelstrom et al. [27] found that the main function of these cells is to differentiate into astrocytes and oligodendrocytes, further forming a glial scar that inhibits lesion expansion and remyelination; some of these cells are also strip cells that participate in the formation of glial scars. Some researchers have transplanted these cells into the lesions of the injured spinal cord of an animal model of hemiplegia and observed a significant improvement in the motor function of the animal [44]. These reports offer hope for noninvasive cell therapy, as these endogenous cells are able to be directly activated to function without the need for traumatic cell transplantation. According to Nomura et al. [44], radial glial cells support and guide axonal regeneration. In contrast, Chang [99] concluded that the key to improving the symptoms of SCI after radial glial cell transplantation is the radial colloid. Cells are able to inhibit inflammatory cells and activate endogenous stem cells [100]. Studies have also generated neurons using in situ glial cell reprogramming to achieve spinal cord function recovery [101]. Su et al. [102] used gene transfer to introduce SOX2 into damaged spinal glial cells and administered histidine deacetylase and valproic acid to reprogram them into neurons. After 14 weeks, the motor function of the rats was significantly improved. Therefore, an in-depth understanding of SCI pathological processes and the molecular mechanisms regulating these processes is very important for the identification of potential targeted therapies and to effectively promote spinal cord regeneration and functional recovery. In theory, although the application of AESCs to treat SCI has obvious advantages, some uncertainties and limitations exist, and thus many controversies regarding their clinical applications persist. Based on the uniqueness of AESCs, their potential as a clinical treatment for SCI is more advantageous than other types of stem cells. Therefore, the future clinical application of AESCs will be a hot topic.

### Spermatogonial stem cells

Spermatogonial stem cells (SSCs) are a subtype of spermatogonia [37] that maintain the spermatozoa throughout the life of the organism. These cells possess self-replication and self-renewal abilities and are the only type of stem cell that is able to pass the genetic material to the offspring. In recent years, with the rapid development of regenerative medicine and translational medicine, SSCs are no longer only producing sperm and assisting in reproduction in the traditional sense but are further expanded based on the original study. SSCs have been cultured in vitro to obtain pluripotent embryonic stem cells with the potential to differentiate into multiple lineages of cells [37, 40]. Compared with the aforementioned stem cells, SSCs have the advantages of being produced throughout the lifetime and a lack of ethical problems, tumorigenicity, and immune rejection. According to Glaser et al. [38], adult mouse testicular tissue germ-line stem cells can be transdifferentiated into NSCs under special induction conditions, and these cells can be further induced to produce functional GABAergic neurons, glutamatergic neurons, serotonergic neurons, and glial cells. Thus, SSCs have the potential to differentiate into various cells of the nervous system. Subsequently, Streckfuss et al. [39] transdifferentiated mouse SSCs into neurons and glial cells of different subtypes with appropriate physiological functions. These studies laid the foundation for the use of SSCs for transdifferentiation into neurons as a treatment for neurological and neurodegenerative diseases. As shown in the study by Simon et al. [103], SSCs are able to be transplanted into prostate, skin and uterine tissues, and subsequently differentiate into epithelial cells similar to these host tissue. The multidifferentiation potential of SSCs is susceptible to the peripheral system environment. Liu et al. [104] induced rat SSCs to differentiate into dopaminergic neurons in vitro and transplanted them into Parkinson model animals, significantly improving functional deficits in the transplanted animals. Wang et al. [105] confirmed the differentiation of pig SSCs into neuron-like cells and fat cells. Based on these studies, SSCs are expected to be an alternative source of ESCs and NSCs. Although studies have confirmed that SSCs can differentiate into neurons, these studies have undergone intermediate transdifferentiation of NSCs during the process of differentiation to produce neurons [103]. Hao et al. have recently used unique induction conditions to directly transduce rat and mouse SSCs to differentiate into functional dopaminergic neurons and spinal motor neurons; this differentiation process does not require intermediate ESCs or NSCs transition processes [41, 42]. Thus, the associated risks for future clinical applications are reduced. Currently, few studies have investigated SSC transplantation for SCI, and most have focused on neurodegenerative diseases. One recent study by Nazm Bojnordi et al. [43] transdifferentiated SSCs into oligodendrocytes and then transplanted the cells into a demyelinated rat model to observe the formation of myelin, suggesting that SSCs are expected to become the ideal therapeutic cell for the treatment of spinal cord demyelinating lesions. At present, SSC treatment of nervous system diseases is almost exclusively designed to substitute for differentiation, and no reports of secretion of various factors by these cells have been found.

### Induced pluripotent stem cells

As mentioned above, although ESCs might substantially improve the treatment of SCI, their application has many limitations. Therefore, a suitable alternative source is an urgent unmet medical need. Induced pluripotent stem cells (iPSCs) are considered effective alternative cell sources for ESCs that can be obtained from cell reprogramming by introducing transcription factors into somatic cells, as described by Yamanaka. Currently used methods include virus-mediated gene transfection, targeted insertion, gene transposon insertion and protein transfection [106]. Moreover, since somatic cells are typically isolated from the patient’s own cells, this autologous method can prevent the occurrence of immune rejection. Although iPSCs and ESCs have their own cytological advantages, a common drawback is the possibility of producing solid tumors [107], mainly due to their possible pluripotency that is no longer controlled after transplantation. Fortunately, several studies have begun to reduce the tumorigenicity. The generation of nonviral iPSCs, including piggyBac transposons, microRNAs, mRNAs, episomal vectors, small molecule compounds, and recombinant proteins, represents an effective and reproducible alternative [108–114].

NSCs derived from transplanted iPSC promote remyelination, axonal regeneration, and the secretion of neurotrophic factors, while reducing inflammation [35]. According to existing studies, orthotopic transplantation of iPSCs is safe and effective as a treatment for SCI when iPSCs are induced to differentiate into oligodendrocytes or neurons and then retransplanted into a rat, mouse or primate long-tailed monkey spinal contusion model. After 3 to 5 weeks, the motor function defects were significantly improved, and no tumors occurred [115, 116]. Lu and colleagues implanted neural stem cells that had been differentiated from human iPSCs into immunodeficient rats 2 weeks after a C5 half-cut injury. Three months later, the authors discovered that the axons extended nearly completely throughout the entire rostral-to-caudal extent and formed synapses with rodent neurons. Synapses also formed between host axons and iPSCs [117]. Interestingly, this experiment used the iPSCs from an 86-year-old human, indicating that age does not appear to be an obstacle to the expression of highly plastic neural stem cells. As shown in the study by Salewski et al. [118], the transplantation of iPSC-derived neurons into rodent spinal crush injury or contusion models effectively restores motor function in the animal. Jendelova and colleagues observed beneficial effects of iPSC-NPCs on preserving the host tissue, reducing the glial scar, increasing axonal sprouting, and promoting motor functional recovery [119]. Although these studies currently provide large amounts of exciting evidence for the therapeutic effects of iPSCs, some experiments also found that iPSC-NPCs did not improve or even reduced the neurological function score after SCI. This discrepancy may be related to the use of different delivery times and administration routes. In addition, the effectiveness and feasibility of iPSCs in clinical treatments remains to be explored. First, the optimal time for cell transplantation is the subacute phase after SCI, while the assessment of the quality of iPSC-NPCs generated from the patient’s somatic cells may take more than 1 year [6]. Therefore, the idea of establishing a cell bank to store hiPSC-NPCs was proposed [120]. Narihito Nagoshi has collaborated with the Center for iPS Cell Research and Application at Kyoto University, which provides clinical-grade integration-free hiPSC lines. Another problem is that most patients who currently suffer from an SCI are usually in the chronic phase; however, few studies have investigated the use of iPSCs in this stage. Thus, additional studies focusing on the chronic phase should be performed.

## Conclusions

**Fig. 1:**
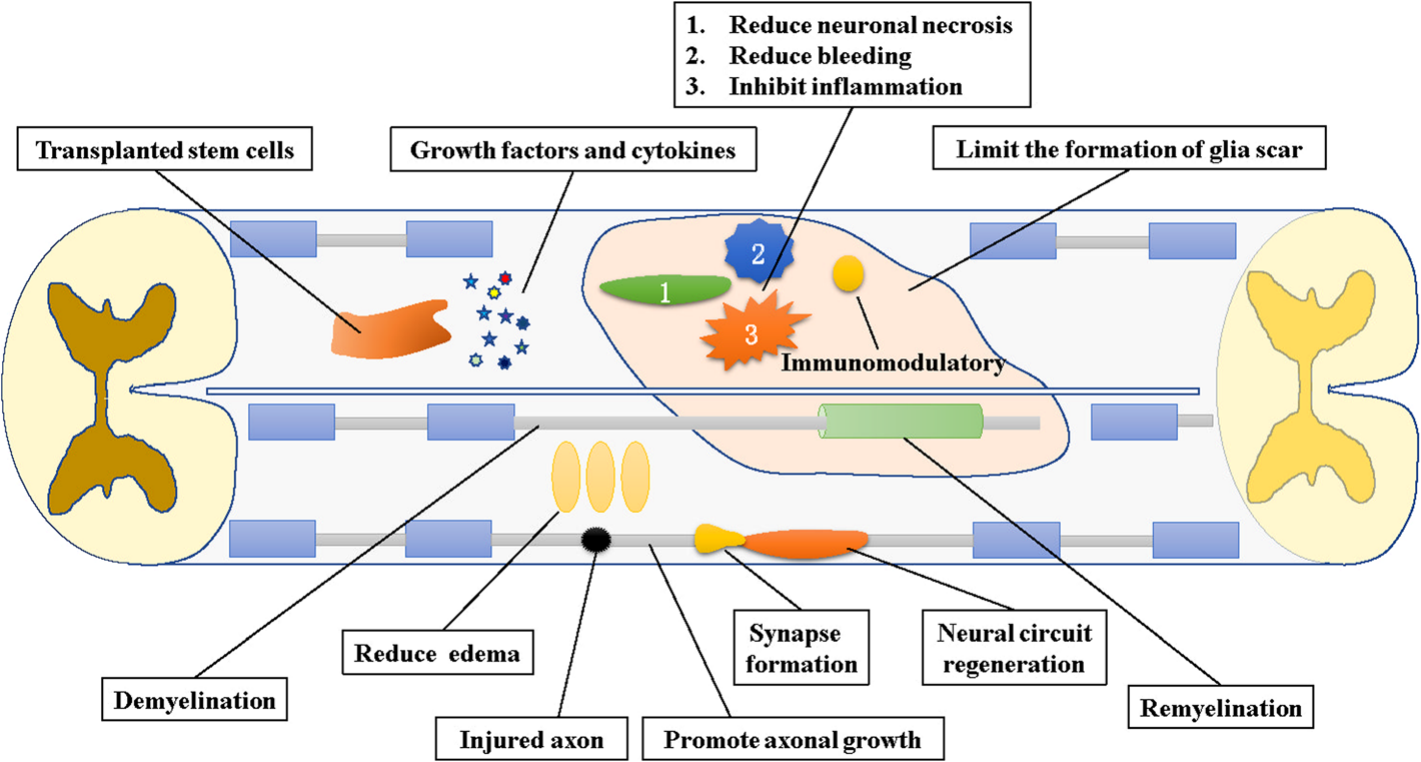
The potential mechanism between stem cell and spinal cord injury

Stem cell therapy has shown great potential in reconstructing the injured spinal cord and promoting functional recovery (Fig. 1), but many problems remain to be resolved before the clinical application of this therapy. ESCs have limited clinical application due to ethical issues regarding their origin and tumorigenicity after transplantation. In contrast, iPSCs are derived from the reprogramming of autologous cells; although these cells are not associated with ethical issues, the efficiency and safety of their clinical applications has been questioned due to the requirement for exogenous gene transfer [121]. MSCs present no ethical issues and have a wide range of sources. They have unique advantages in inhibiting inflammatory cell activation, reconstituting local blood supply systems, and secreting nerve-promoting factors, but their differentiation potential is weaker than their secretory potential; thus, these cells are not the best candidate for the treatment of severe chronic injury in patients with SCI. The biological characteristics of MSCs from different sources are not the same, and these cells are not ideal for the treatment of advanced SCI. Due to the complexity and dynamic variability of the pathological process of SCI, no two SCI injuries are identical. Therefore, clinicians must comprehensively consider the specific conditions of SCI injury and choose an appropriate treatment strategy to achieve the best therapeutic effect. Clinicians must determine which strategy will be the most effective for patients with different types or stages of SCI. Is the first goal to control inflammation, eliminate glial scars, administer a growth factor treatment, reconstitute the blood supply system, supplement the missing neurons, or supplement the oligodendrocytes to reconstruct myelin? Different types of stem cells exert different therapeutic effects. A reasonable treatment plan must be developed based on the pathological condition of the SCI and the characteristics of alternative cells to achieve a better curative effect. Currently, the efficacy of stem cell therapy for SCI is highly controversial because it does not completely achieve the docking of different SCIs with the most suitable transplanted stem cells. In addition to the points mentioned above, the problem that must be carefully considered is that the preclinical study of the therapeutic effectiveness of stem cells at improving various parameters should be adjusted according to the species of the experimental animals. Although the pathology and outcomes of SCI in different species are similar to those in humans, effective cell therapy regimens in rodents and primates may not be effective in humans. In addition, the choice of the optimal time window for SCI is important for selecting which stem cells to use for treatment to achieve therapeutic effects. Therefore, a large number of similar case models and treatment strategies are needed as a basis for repeated practice and demonstration to achieve clinical transformation and applications. In addition, stem cells are not omnipotent. To maximize the effect of stem cell therapy, it is impossible to rely solely on stem cells. A comprehensive treatment plan that also includes the combined application of biological materials and drugs must be used for different SCI cases and pathological processes. Effective treatment and recovery of bodily function are possible with a suitable comprehensive treatment plan.

## Data Availability

Not applicable.

## Abbreviations

AE-MSCs: Amniotic epithelial mesenchymal stem cells
AESCs: Adult endogenous stem cells
A-MSCs: Adipose-derived mesenchymal stem cells
BDNF: Brain-derived neurotrophic factor
bFGF: Basic fibroblast growth factor
BM-MSCs: Bone marrow mesenchymal stem cells
CNS: Central nervous system
EGF: Epidermal growth factor
ESCs: Embryonic stem cells
FGF: Fibroblast growth factor
GDNF: Glial-derived neurotrophic factor
IGF-1: Insulin growth factor-1
IL-1: Interleukin-1
iPSCs: Induced pluripotent stem cells
MSCs: Mesenchymal stem cells
NCAM: Neural cell adhesion molecule
NGF: Nerve growth factor
NPCs: Neural precursor cells
NSCs: Neural stem cells
NT-3: Neurotrophin-3
OPCs: Oligodendrocyte precursor cells
SCI: Spinal cord injury
SHH: Sonic hedgehog
SSCs: Spermatogonial stem cells
UC-MSCs: Umbilical cord mesenchymal stem cells
VEGF: Vascular endothelial growth factor

## References

[1] Tator CH, Fehlings MG (1991). Review of the secondary injury theory of acute spinal cord trauma with emphasis on vascular mechanisms. J Neurosurg.

[2] Guertin PA (2019). A central pattern generator in the spinal cord for the central control of micturition: an opportunity for first-in-class drug treatments. Asia Pac J Clin Trials Nerv Syst Dis.

[3] Silver J, Miller JH (2004). Regeneration beyond the glial scar. Nat Rev Neurosci.

[4] Donnelly DJ, Popovich PG (2008). Inflammation and its role in neuroprotection, axonal regeneration and functional recovery after spinal cord injury. Exp Neurol.

[5] Badner A, Siddiqui AM, Fehlings MG (2017). Spinal cord injuries: how could cell therapy help?. Expert Opin Biol Ther.

[6] Nagoshi N, Okano H (2017). Applications of induced pluripotent stem cell technologies in spinal cord injury. J Neurochem.

[7] Orlic D, Kajstura J, Chimenti S, Bodine DM, Leri A, Anversa P (2003). Bone marrow stem cells regenerate infarcted myocardium. Pediatr Transplant.

[8] Lovell-Badge R (2001). The future for stem cell research. Nature.

[9] Neves J, Sousa-Victor P, Jasper H (2017). Rejuvenating strategies for stem cell-based therapies in aging. Cell Stem Cell.

[10] Dang PN, Dwivedi N, Phillips LM, Yu X, Herberg S, Bowerman C, Solorio LD, Murphy WL, Alsberg E (2016). Controlled dual growth factor delivery from microparticles incorporated within human bone marrow-derived mesenchymal stem cell aggregates for enhanced bone tissue engineering via endochondral ossification. Stem Cells Transl Med.

[11] Silva NA, Sousa N, Reis RL, Salgado AJ (2014). From basics to clinical: a comprehensive review on spinal cord injury. Prog Neurobiol.

[12] Vismara I, Papa S, Rossi F, Forloni G, Veglianese P (2017). Current options for cell therapy in spinal cord injury. Trends Mol Med.

[13] Dasari VR, Veeravalli KK, Dinh DH (2014). Mesenchymal stem cells in the treatment of spinal cord injuries: a review. World J Stem Cells.

[14] Honmou O, Houkin K, Matsunaga T, Niitsu Y, Ishiai S, Onodera R, Waxman SG, Kocsis JD (2011). Intravenous administration of auto serum-expanded autologous mesenchymal stem cells in stroke. Brain.

[15] Cofano Fabio, Boido Marina, Monticelli Matteo, Zenga Francesco, Ducati Alessandro, Vercelli Alessandro, Garbossa Diego (2019). Mesenchymal Stem Cells for Spinal Cord Injury: Current Options, Limitations, and Future of Cell Therapy. International Journal of Molecular Sciences.

[16] Lee MW, Yang MS, Park JS, Kim HC, Kim YJ, Choi J (2005). Isolation of mesenchymal stem cells from cryopreserved human umbilical cord blood. Int J Hematol.

[17] Evans MJ, Kaufman MH (1981). Establishment in culture of pluripotential cells from mouse embryos. Nature.

[18] Shroff G, Dhanda Titus J, Shroff R (2017). A review of the emerging potential therapy for neurological disorders: human embryonic stem cell therapy. Am J Stem Cells.

[19] Harper JM, Krishnan C, Darman JS, Deshpande DM, Peck S, Shats I, Backovic S, Rothstein JD, Kerr DA (2004). Axonal growth of embryonic stem cell-derived motoneurons in vitro and in motoneuron-injured adult rats. Proc Natl Acad Sci U S A.

[20] Iwai H, Shimada H, Nishimura S, Kobayashi Y, Itakura G, Hori K, Hikishima K, Ebise H, Negishi N, Shibata S, Habu S, Toyama Y, Nakamura M, Okano H (2015). Allogeneic neural stem/progenitor cells derived from embryonic stem cells promote functional recovery after transplantation into injured spinal cord of nonhuman primates. Stem Cells Transl Med.

[21] Yang JR, Liao CH, Pang CY, Huang LL, Chen YL, Shiue YL, Chen LR (2013). Transplantation of porcine embryonic stem cells and their derived neuronal progenitors in a spinal cord injury rat model. Cytotherapy.

[22] Johnson PJ, Tatara A, Shiu A, Sakiyama-Elbert SE (2010). Controlled release of neurotrophin-3 and platelet-derived growth factor from fibrin scaffolds containing neural progenitor cells enhances survival and differentiation into neurons in a subacute model of SCI. Cell Transplant.

[23] Keirstead HS, Nistor G, Bernal G, Totoiu M, Cloutier F, Sharp K, Steward O (2005). Human embryonic stem cell-derived oligodendrocyte progenitor cell transplants remyelinate and restore locomotion after spinal cord injury. J Neurosci.

[24] Tetzlaff W, Okon EB, Karimi-Abdolrezaee S, Hill CE, Sparling JS, Plemel JR, Plunet WT, Tsai EC, Baptiste D, Smithson LJ, Kawaja MD, Fehlings MG, Kwon BK (2011). A systematic review of cellular transplantation therapies for spinal cord injury. J Neurotrauma.

[25] Nakamura M, Okano H (2013). Cell transplantation therapies for spinal cord injury focusing on induced pluripotent stem cells. Cell Res.

[26] Iyer NR, Wilems TS, Sakiyama-Elbert SE (2017). Stem cells for spinal cord injury: strategies to inform differentiation and transplantation. Biotechnol Bioeng.

[27] Sabelstrom H, Stenudd M, Frisen J (2014). Neural stem cells in the adult spinal cord. Exp Neurol.

[28] Gregoire CA, Goldenstein BL, Floriddia EM, Barnabe-Heider F, Fernandes KJ (2015). Endogenous neural stem cell responses to stroke and spinal cord injury. Glia.

[29] Lu P, Wang Y, Graham L, McHale K, Gao M, Wu D, Brock J, Blesch A, Rosenzweig ES, Havton LA, Zheng B, Conner JM, Marsala M, Tuszynski MH (2012). Long-distance growth and connectivity of neural stem cells after severe spinal cord injury. Cell.

[30] Kerr CL, Letzen BS, Hill CM, Agrawal G, Thakor NV, Sterneckert JL, Gearhart JD, All AH (2010). Efficient differentiation of human embryonic stem cells into oligodendrocyte progenitors for application in a rat contusion model of spinal cord injury. Int J Neurosci.

[31] Hofstetter CP, Holmstrom NA, Lilja JA, Schweinhardt P, Hao J, Spenger C, Wiesenfeld-Hallin Z, Kurpad SN, Frisen J, Olson L (2005). Allodynia limits the usefulness of intraspinal neural stem cell grafts; directed differentiation improves outcome. Nat Neurosci.

[32] Gabel BC, Curtis EI, Marsala M, Ciacci JD (2017). A review of stem cell therapy for spinal cord injury: large animal models and the frontier in humans. World Neurosurg.

[33] Ahuja CS, Wilson JR, Nori S, Kotter MRN, Druschel C, Curt A, Fehlings MG (2017). Traumatic spinal cord injury. Nat Rev Dis Primers.

[34] Pereira Inês M., Marote Ana, Salgado António J., Silva Nuno A. (2019). Filling the Gap: Neural Stem Cells as A Promising Therapy for Spinal Cord Injury. Pharmaceuticals.

[35] Yousefifard M, Rahimi-Movaghar V, Nasirinezhad F, Baikpour M, Safari S, Saadat S, Moghadas Jafari A, Asady H, Razavi Tousi SM, Hosseini M (2016). Neural stem/progenitor cell transplantation for spinal cord injury treatment; a systematic review and meta-analysis. Neuroscience.

[36] Deng J, Zhang Y, Xie Y, Zhang L, Tang P (2018). Cell transplantation for spinal cord injury: tumorigenicity of induced pluripotent stem cell-derived neural stem/progenitor cells. Stem Cells Int.

[37] Kanatsu-Shinohara M, Shinohara T (2013). Spermatogonial stem cell self-renewal and development. Annu Rev Cell Dev Biol.

[38] Glaser T, Opitz T, Kischlat T, Konang R, Sasse P, Fleischmann BK, Engel W, Nayernia K, Brustle O (2008). Adult germ line stem cells as a source of functional neurons and glia. Stem Cells.

[39] Streckfuss-Bomeke K, Vlasov A, Hulsmann S, Yin D, Nayernia K, Engel W, Hasenfuss G, Guan K (2009). Generation of functional neurons and glia from multipotent adult mouse germ-line stem cells. Stem Cell Res.

[40] Kossack N, Meneses J, Shefi S, Nguyen HN, Chavez S, Nicholas C, Gromoll J, Turek PJ, Reijo-Pera RA (2009). Isolation and characterization of pluripotent human spermatogonial stem cell-derived cells. Stem Cells.

[41] Yang H, Liu Y, Hai Y, Guo Y, Yang S, Li Z, Gao WQ, He Z (2015). Efficient conversion of spermatogonial stem cells to phenotypic and functional dopaminergic neurons via the PI3K/Akt and P21/Smurf2/Nolz1 pathway. Mol Neurobiol.

[42] Yang H, Liu C, Chen B, An J, Zhang R, Zhang Q, Zhao J, He B, Hao DJ (2017). Efficient generation of functionally active spinal cord neurons from spermatogonial stem cells. Mol Neurobiol.

[43] Nazm Bojnordi M, Movahedin M, Tiraihi T, Javan M, Ghasemi Hamidabadi H (2014). Oligoprogenitor cells derived from spermatogonia stem cells improve remyelination in demyelination model. Mol Biotechnol.

[44] Nomura H, Kim H, Mothe A, Zahir T, Kulbatski I, Morshead CM, Shoichet MS, Tator CH (2010). Endogenous radial glial cells support regenerating axons after spinal cord transection. Neuroreport.

[45] Panayiotou E, Malas S (2013). Adult spinal cord ependymal layer: a promising pool of quiescent stem cells to treat spinal cord injury. Front Physiol.

[46] Liu Y, Tan B, Wang L, Long Z, Li Y, Liao W, Wu Y (2015). Endogenous neural stem cells in central canal of adult rats acquired limited ability to differentiate into neurons following mild spinal cord injury. Int J Clin Exp Pathol.

[47] Ahuja CS, Fehlings M (2016). Concise review: bridging the gap: novel neuroregenerative and neuroprotective strategies in spinal cord injury. Stem Cells Transl Med.

[48] Xue PXZF, Kou YH, Han S, Wang TB, Zhang DY, Jiang BG (2017). Pre-hospital and in-hospital first aid programs and specifications for spine and spinal cord injury in Beijing, China: study protocol for a prospective, multicenter, nonrandomized controlled trial. Asia Pac J Clin Trials Nerv Syst Dis.

[49] Mukaino M, Nakamura M, Yamada O, Okada S, Morikawa S, Renault-Mihara F, Iwanami A, Ikegami T, Ohsugi Y, Tsuji O, Katoh H, Matsuzaki Y, Toyama Y, Liu M, Okano H (2010). Anti-IL-6-receptor antibody promotes repair of spinal cord injury by inducing microglia-dominant inflammation. Exp Neurol.

[50] Anwar MA, Al Shehabi TS, Eid AH (2016). Inflammogenesis of secondary spinal cord injury. Front Cell Neurosci.

[51] Gao L, Zhang Z, Xu W, Li T, Ying G, Qin B, Li J, Zheng J, Zhao T, Yan F, Zhu Y, Chen G (2019). Natrium benzoate alleviates neuronal apoptosis via the DJ-1-related anti-oxidative stress pathway involving Akt phosphorylation in a rat model of traumatic spinal cord injury. Front Mol Neurosci.

[52] Sugawara T, Chan PH (2003). Reactive oxygen radicals and pathogenesis of neuronal death after cerebral ischemia. Antioxid Redox Signal.

[53] Wang X, Wu X, Liu Q, Kong G, Zhou J, Jiang J, Wu X, Huang Z, Su W, Zhu Q (2017). Ketogenic metabolism inhibits histone deacetylase (HDAC) and reduces oxidative stress after spinal cord injury in rats. Neuroscience.

[54] Zhou L, Ouyang L, Lin S, Chen S, Liu Y, Zhou W, Wang X (2018). Protective role of beta-carotene against oxidative stress and neuroinflammation in a rat model of spinal cord injury. Int Immunopharmacol.

[55] Shimizu EN, Seifert JL, Johnson KJ, Romero-Ortega MI (2018). Prophylactic riluzole attenuates oxidative stress damage in spinal cord distraction. J Neurotrauma.

[56] Zhang L, Kaneko S, Kikuchi K, Sano A, Maeda M, Kishino A, Shibata S, Mukaino M, Toyama Y, Liu M, Kimura T, Okano H, Nakamura M (2014). Rewiring of regenerated axons by combining treadmill training with semaphorin3A inhibition. Mol Brain.

[57] Tashiro S, Nishimura S, Iwai H, Sugai K, Zhang L, Shinozaki M, Iwanami A, Toyama Y, Liu M, Okano H, Nakamura M (2016). Functional recovery from neural stem/progenitor cell transplantation combined with treadmill training in mice with chronic spinal cord injury. Sci Rep.

[58] Assinck P, Duncan GJ, Hilton BJ, Plemel JR, Tetzlaff W (2017). Cell transplantation therapy for spinal cord injury. Nat Neurosci.

[59] Emgard M, Piao J, Aineskog H, Liu J, Calzarossa C, Odeberg J, Holmberg L, Samuelsson EB, Bezubik B, Vincent PH, Falci SP, Seiger A, Akesson E, Sundstrom E (2014). Neuroprotective effects of human spinal cord-derived neural precursor cells after transplantation to the injured spinal cord. Exp Neurol.

[60] Hawryluk GW, Mothe A, Wang J, Wang S, Tator C, Fehlings MG (2012). An in vivo characterization of trophic factor production following neural precursor cell or bone marrow stromal cell transplantation for spinal cord injury. Stem Cells Dev.

[61] Kadoya K, Lu P, Nguyen K, Lee-Kubli C, Kumamaru H, Yao L, Knackert J, Poplawski G, Dulin JN, Strobl H, Takashima Y, Biane J, Conner J, Zhang SC, Tuszynski MH (2016). Spinal cord reconstitution with homologous neural grafts enables robust corticospinal regeneration. Nat Med.

[62] Armstrong RJ, Hurelbrink CB, Tyers P, Ratcliffe EL, Richards A, Dunnett SB, Rosser AE, Barker RA (2002). The potential for circuit reconstruction by expanded neural precursor cells explored through porcine xenografts in a rat model of Parkinson’s disease. Exp Neurol.

[63] Bregman BS, Kunkel-Bagden E, Reier PJ, Dai HN, McAtee M, Gao D (1993). Recovery of function after spinal cord injury: mechanisms underlying transplant-mediated recovery of function differ after spinal cord injury in newborn and adult rats. Exp Neurol.

[64] Stenudd M, Sabelstrom H, Frisen J (2015). Role of endogenous neural stem cells in spinal cord injury and repair. JAMA Neurol.

[65] Giusto E, Donega M, Cossetti C, Pluchino S (2014). Neuro-immune interactions of neural stem cell transplants: from animal disease models to human trials. Exp Neurol.

[66] Volkman R, Offen D (2017). Concise review: mesenchymal stem cells in neurodegenerative diseases. Stem Cells.

[67] Qu J, Zhang H (2017). Roles of mesenchymal stem cells in spinal cord injury. Stem Cells Int.

[68] DeBrot A, Yao L (2018). The combination of induced pluripotent stem cells and bioscaffolds holds promise for spinal cord regeneration. Neural Regen Res.

[69] Lee RH, Kim B, Choi I, Kim H, Choi HS, Suh K, Bae YC, Jung JS (2004). Characterization and expression analysis of mesenchymal stem cells from human bone marrow and adipose tissue. Cell Physiol Biochem.

[70] Cho SR, Kim YR, Kang HS, Yim SH, Park CI, Min YH, Lee BH, Shin JC, Lim JB (2016). Functional recovery after the transplantation of neurally differentiated mesenchymal stem cells derived from bone marrow in a rat model of spinal cord injury. Cell Transplant.

[71] Luo H, Xu C, Liu Z, Yang L, Hong Y, Liu G, Zhong H, Cai X, Lin X, Chen X, Wang C, Nanwen Z, Xu W (2019). Neural differentiation of bone marrow mesenchymal stem cells with human brain-derived neurotrophic factor gene-modified in functionalized self-assembling peptide hydrogel in vitro. J Cell Biochem.

[72] Osaka M, Honmou O, Murakami T, Nonaka T, Houkin K, Hamada H, Kocsis JD (2010). Intravenous administration of mesenchymal stem cells derived from bone marrow after contusive spinal cord injury improves functional outcome. Brain Res.

[73] Ramalho BDS, Almeida FM, Sales CM, de Lima S, Martinez AMB (2018). Injection of bone marrow mesenchymal stem cells by intravenous or intraperitoneal routes is a viable alternative to spinal cord injury treatment in mice. Neural Regen Res.

[74] Neirinckx V, Cantinieaux D, Coste C, Rogister B, Franzen R, Wislet-Gendebien S (2014). Concise review: spinal cord injuries: how could adult mesenchymal and neural crest stem cells take up the challenge?. Stem Cells.

[75] Nakajima H, Uchida K, Guerrero AR, Watanabe S, Sugita D, Takeura N, Yoshida A, Long G, Wright KT, Johnson WE, Baba H (2012). Transplantation of mesenchymal stem cells promotes an alternative pathway of macrophage activation and functional recovery after spinal cord injury. J Neurotrauma.

[76] Salgado AJ, Reis RL, Sousa NJ, Gimble JM (2010). Adipose tissue derived stem cells secretome: soluble factors and their roles in regenerative medicine. Curr Stem Cell Res Ther.

[77] Kim Y, Jo SH, Kim WH, Kweon OK (2015). Antioxidant and anti-inflammatory effects of intravenously injected adipose derived mesenchymal stem cells in dogs with acute spinal cord injury. Stem Cell Res Ther.

[78] Kolar MK, Kingham PJ, Novikova LN, Wiberg M, Novikov LN (2014). The therapeutic effects of human adipose-derived stem cells in a rat cervical spinal cord injury model. Stem Cells Dev.

[79] Kokai LE, Marra K, Rubin JP (2014). Adipose stem cells: biology and clinical applications for tissue repair and regeneration. Transl Res.

[80] Kim Y, Lee SH, Kim WH, Kweon OK (2016). Transplantation of adipose derived mesenchymal stem cells for acute thoracolumbar disc disease with no deep pain perception in dogs. J Vet Sci.

[81] Zhou J, Lu P, Ren H, Zheng Z, Ji J, Liu H, Jiang F, Ling S, Heng BC, Hu X, Ouyang H (2014). 17beta-estradiol protects human eyelid-derived adipose stem cells against cytotoxicity and increases transplanted cell survival in spinal cord injury. J Cell Mol Med.

[82] Hyun J, Grova M, Nejadnik H, Lo D, Morrison S, Montoro D, Chung M, Zimmermann A, Walmsley GG, Lee M, Daldrup-Link H, Wan DC, Longaker MT (2013). Enhancing in vivo survival of adipose-derived stromal cells through Bcl-2 overexpression using a minicircle vector. Stem Cells Transl Med.

[83] Lee SH, Kim Y, Rhew D, Kuk M, Kim M, Kim WH, Kweon OK (2015). Effect of the combination of mesenchymal stromal cells and chondroitinase ABC on chronic spinal cord injury. Cytotherapy.

[84] Hur JW, Cho TH, Park DH, Lee JB, Park JY, Chung YG (2016). Intrathecal transplantation of autologous adipose-derived mesenchymal stem cells for treating spinal cord injury: a human trial. J Spinal Cord Med.

[85] Sanluis-Verdes A, Sanluis-Verdes N, Manso-Revilla MJ, Castro-Castro AM, Pombo-Otero J, Fraga-Marino M, Sanchez-Ibanez J, Domenech N, Rendal-Vazquez ME (2017). Tissue engineering for neurodegenerative diseases using human amniotic membrane and umbilical cord. Cell Tissue Bank.

[86] Caron I, Rossi F, Papa S, Aloe R, Sculco M, Mauri E, Sacchetti A, Erba E, Panini N, Parazzi V, Barilani M, Forloni G, Perale G, Lazzari L, Veglianese P (2016). A new three dimensional biomimetic hydrogel to deliver factors secreted by human mesenchymal stem cells in spinal cord injury. Biomaterials.

[87] Bottai D, Scesa G, Cigognini D, Adami R, Nicora E, Abrignani S, Di Giulio AM, Gorio A (2014). Third trimester NG2-positive amniotic fluid cells are effective in improving repair in spinal cord injury. Exp Neurol.

[88] Gao S, Ding J, Xiao HJ, Li ZQ, Chen Y, Zhou XS, Wang JE, Wu J, Shi WZ (2014). Anti-inflammatory and anti-apoptotic effect of combined treatment with methylprednisolone and amniotic membrane mesenchymal stem cells after spinal cord injury in rats. Neurochem Res.

[89] Sankar V, Muthusamy R (2003). Role of human amniotic epithelial cell transplantation in spinal cord injury repair research. Neuroscience.

[90] Liu CB, Huang H, Sun P, Ma SZ, Liu AH, Xue J, Fu JH, Liang YQ, Liu B, Wu DY, Lu SH, Zhang XZ (2016). Human umbilical cord-derived mesenchymal stromal cells improve left ventricular function, perfusion, and remodeling in a porcine model of chronic myocardial ischemia. Stem Cells Transl Med.

[91] Park SE, Jung NY, Lee NK, Lee J, Hyung B, Myeong SH, Kim HS, Suh YL, Lee JI, Cho KR, Kim DH, Choi SJ, Chang JW, Na DL (2016). Distribution of human umbilical cord blood-derived mesenchymal stem cells (hUCB-MSCs) in canines after intracerebroventricular injection. Neurobiol Aging.

[92] Kang KS, Kim SW, Oh YH, Yu JW, Kim KY, Park HK, Song CH, Han H (2005). A 37-year-old spinal cord-injured female patient, transplanted of multipotent stem cells from human UC blood, with improved sensory perception and mobility, both functionally and morphologically: a case study. Cytotherapy.

[93] Yao L, He C, Zhao Y, Wang J, Tang M, Li J, Wu Y, Ao L, Hu X (2013). Human umbilical cord blood stem cell transplantation for the treatment of chronic spinal cord injury: electrophysiological changes and long-term efficacy. Neural Regen Res.

[94] Zhu H, Poon W, Liu Y, Leung GK, Wong Y, Feng Y, Ng SCP, Tsang KS, Sun DTF, Yeung DK, Shen C, Niu F, Xu Z, Tan P, Tang S, Gao H, Cha Y, So KF, Fleischaker R, Sun D, Chen J, Lai J, Cheng W, Young W (2016). Phase I-II clinical trial assessing safety and efficacy of umbilical cord blood mononuclear cell transplant therapy of chronic complete spinal cord injury. Cell Transplant.

[95] Guo L, Rolfe AJ, Wang X, Tai W, Cheng Z, Cao K, Chen X, Xu Y, Sun D, Li J, He X, Young W, Fan J, Ren Y (2016). Rescuing macrophage normal function in spinal cord injury with embryonic stem cell conditioned media. Mol Brain.

[96] Salewski RP, Mitchell RA, Shen C, Fehlings MG (2015). Transplantation of neural stem cells clonally derived from embryonic stem cells promotes recovery after murine spinal cord injury. Stem Cells Dev.

[97] Koch P, Opitz T, Steinbeck JA, Ladewig J, Brustle O (2009). A rosette-type, self-renewing human ES cell-derived neural stem cell with potential for in vitro instruction and synaptic integration. Proc Natl Acad Sci U S A.

[98] Shin S, Mitalipova M, Noggle S, Tibbitts D, Venable A, Rao R, Stice SL (2006). Long-term proliferation of human embryonic stem cell-derived neuroepithelial cells using defined adherent culture conditions. Stem Cells.

[99] Chang YW, Goff LA, Li H, Kane-Goldsmith N, Tzatzalos E, Hart RP, Young W, Grumet M (2009). Rapid induction of genes associated with tissue protection and neural development in contused adult spinal cord after radial glial cell transplantation. J Neurotrauma.

[100] Moreno-Manzano V, Rodriguez-Jimenez FJ, Garcia-Rosello M, Lainez S, Erceg S, Calvo MT, Ronaghi M, Lloret M, Planells-Cases R, Sanchez-Puelles JM, Stojkovic M (2009). Activated spinal cord ependymal stem cells rescue neurological function. Stem Cells.

[101] Chen G, Wernig M, Berninger B, Nakafuku M, Parmar M, Zhang CL (2015). In vivo reprogramming for brain and spinal cord repair, eNeuro.

[102] Su Z, Niu W, Liu ML, Zou Y, Zhang CL (2014). In vivo conversion of astrocytes to neurons in the injured adult spinal cord. Nat Commun.

[103] Simon L, Ekman GC, Kostereva N, Zhang Z, Hess RA, Hofmann MC, Cooke PS (2009). Direct transdifferentiation of stem/progenitor spermatogonia into reproductive and nonreproductive tissues of all germ layers. Stem Cells.

[104] Liu T, Gong Z, Hou L, Huang Y (2012). The induction of rat spermatogonial stem cells into neuronal-like cells and behavioral recovery following transplantation in a rat Parkinson's disease model. Int J Mol Med.

[105] Wang X, Chen T, Zhang Y, Li B, Xu Q, Song C (2015). Isolation and culture of pig spermatogonial stem cells and their in vitro differentiation into neuron-like cells and adipocytes. Int J Mol Sci.

[106] Khazaei M, Ahuja CS, Fehlings MG (2016). Induced pluripotent stem cells for traumatic spinal cord injury. Front Cell Dev Biol.

[107] Goulao M, Lepore AC (2016). iPS cell transplantation for traumatic spinal cord injury. Curr Stem Cell Res Ther.

[108] Woltjen K, Michael IP, Mohseni P, Desai R, Mileikovsky M, Hamalainen R, Cowling R, Wang W, Liu P, Gertsenstein M, Kaji K, Sung HK, Nagy A (2009). piggyBac transposition reprograms fibroblasts to induced pluripotent stem cells. Nature.

[109] Yu J, Hu K, Smuga-Otto K, Tian S, Stewart R, Slukvin II, Thomson JA (2009). Human induced pluripotent stem cells free of vector and transgene sequences. Science.

[110] Kim D, Kim CH, Moon JI, Chung YG, Chang MY, Han BS, Ko S, Yang E, Cha KY, Lanza R, Kim KS (2009). Generation of human induced pluripotent stem cells by direct delivery of reprogramming proteins. Cell Stem Cell.

[111] Warren L, Manos PD, Ahfeldt T, Loh YH, Li H, Lau F, Ebina W, Mandal PK, Smith ZD, Meissner A, Daley GQ, Brack AS, Collins JJ, Cowan C, Schlaeger TM, Rossi DJ (2010). Highly efficient reprogramming to pluripotency and directed differentiation of human cells with synthetic modified mRNA. Cell Stem Cell.

[112] Subramanyam D, Lamouille S, Judson RL, Liu JY, Bucay N, Derynck R, Blelloch R (2011). Multiple targets of miR-302 and miR-372 promote reprogramming of human fibroblasts to induced pluripotent stem cells. Nat Biotechnol.

[113] Huangfu D, Maehr R, Guo W, Eijkelenboom A, Snitow M, Chen AE, Melton DA (2008). Induction of pluripotent stem cells by defined factors is greatly improved by small-molecule compounds. Nat Biotechnol.

[114] Clancy JL, Patel HR, Hussein SM, Tonge PD, Cloonan N, Corso AJ, Li M, Lee DS, Shin JY, Wong JJ, Bailey CG, Benevento M, Munoz J, Chuah A, Wood D, Rasko JE, Heck AJ, Grimmond SM, Rogers IM, Seo JS, Wells CA, Puri MC, Nagy A, Preiss T (2014). Small RNA changes en route to distinct cellular states of induced pluripotency. Nat Commun.

[115] Kawabata S, Takano M, Numasawa-Kuroiwa Y, Itakura G, Kobayashi Y, Nishiyama Y, Sugai K, Nishimura S, Iwai H, Isoda M, Shibata S, Kohyama J, Iwanami A, Toyama Y, Matsumoto M, Nakamura M, Okano H (2016). Grafted human iPS cell-derived oligodendrocyte precursor cells contribute to robust remyelination of demyelinated axons after spinal cord injury. Stem Cell Reports.

[116] All AH, Gharibani P, Gupta S, Bazley FA, Pashai N, Chou BK, Shah S, Resar LM, Cheng L, Gearhart JD, Kerr CL (2015). Early intervention for spinal cord injury with human induced pluripotent stem cells oligodendrocyte progenitors. PLoS One.

[117] Lu P, Woodruff G, Wang Y, Graham L, Hunt M, Wu D, Boehle E, Ahmad R, Poplawski G, Brock J, Goldstein LS, Tuszynski MH (2014). Long-distance axonal growth from human induced pluripotent stem cells after spinal cord injury. Neuron.

[118] Salewski RP, Mitchell RA, Li L, Shen C, Milekovskaia M, Nagy A, Fehlings MG (2015). Transplantation of induced pluripotent stem cell-derived neural stem cells mediate functional recovery following thoracic spinal cord injury through remyelination of axons. Stem Cells Transl Med.

[119] Ruzicka J, Machova-Urdzikova L, Gillick J, Amemori T, Romanyuk N, Karova K, Zaviskova K, Dubisova J, Kubinova S, Murali R, Sykova E, Jhanwar-Uniyal M, Jendelova P (2017). A comparative study of three different types of stem cells for treatment of rat spinal cord injury. Cell Transplant.

[120] Okano H, Yamanaka S (2014). iPS cell technologies: significance and applications to CNS regeneration and disease. Mol Brain.

[121] Tsuji O, Miura K, Okada Y, Fujiyoshi K, Mukaino M, Nagoshi N, Kitamura K, Kumagai G, Nishino M, Tomisato S, Higashi H, Nagai T, Katoh H, Kohda K, Matsuzaki Y, Yuzaki M, Ikeda E, Toyama Y, Nakamura M, Yamanaka S, Okano H (2010). Therapeutic potential of appropriately evaluated safe-induced pluripotent stem cells for spinal cord injury. Proc Natl Acad Sci U S A.

